# Randomized controlled trials for comparison of laparoscopic versus conventional open catheter placement in peritoneal dialysis patients: a meta-analysis

**DOI:** 10.1186/s12882-020-01724-w

**Published:** 2020-02-24

**Authors:** Mei-Lan Sun, Yong Zhang, Bo Wang, Te-An Ma, Hong Jiang, Shou-Liang Hu, Piao Zhang, Yan-Hong Tuo

**Affiliations:** 1 Department of Blood Purification Center, The First Affiliated Hospital of Yangtze University, Jingzhou, Hubei China; 2 Department of Nephrology, Jianli People’s Hospital, Jingzhou, Hubei China; 3 Department of Ultrasonic Imaging, Affiliated Renhe Hospital of China Three Gorges University, Yichang, Hubei China; 4 Department of Nephrology, The First Affiliated Hospital of Yangtze University, Jingzhou, Hubei China; 5 Department of Nephrology, Nanjing General Hospital of Nanjing Military Command, Nanjing, Jiangsu China; 6 Department of Nephrology, The Central Hospital of Wuhan, Tongji Medical College, Huazhong University of Science and Technology, Wuhan, Hubei China

**Keywords:** Laparoscopic catheter placement, Conventional open catheter placement, Peritoneal dialysis, Complications, Meta-analysis, Mei-Lan sun and Yong Zhang are contributed equally to this work

## Abstract

**Background:**

The application of laparoscopic catheterization technology in peritoneal dialysis (PD) patients has recently increased. However, the advantages and disadvantages of laparoscopic versus conventional open PD catheter placement are still controversial. The aim of this meta-analysis is to assess the complications of catheterization in PD patients and to provide a reference for choosing a PD-catheter placement technique in the clinic.

**Methods:**

We searched numerous databases, including Embase, PubMed, CNKI and the Cochrane Library, for published randomized controlled trials (RCTs).

**Results:**

Eight relevant studies (*n* = 646) were included in the meta-analysis. The pooled results showed a lower incidence of catheter migration (OR: 0.42, 95% CI: 0.19 to 0.90, P: 0.03) and catheter removal (OR: 0.41, 95% CI: 0.21 to 0.79, P: 0.008) but a higher incidence of bleeding (OR: 3.25, 95% CI: 1.18 to 8.97, P: 0.02) with a laparoscopic approach than with a conventional approach. There was no significant difference in the incidence of omentum adhesion (OR: 0.32, 95% CI: 0.05 to 2.10, P: 0.24), hernia (OR: 0.38, 95% CI: 0.09 to 1.68, P: 0.20), leakage (OR: 0.69, 95% CI: 0.38 to 1.26, P: 0.23), intestinal obstruction (OR: 0.96, 95% CI: 0.48 to 1.91, P: 0.90) or perforation (OR: 0.95, 95% CI: 0.06 to 15.42, P: 0.97). The statistical analysis showed no significant difference in early (OR: 0.44, 95% CI: 0.15 to 1.33, P: 0.15), late (OR: 0.89, 95% CI: 0.41 to 1.90, P: 0.76) or total (OR: 0.68, 95% CI: 0.42 to 1.12, P: 0.13) peritonitis infections between the 2 groups, and there are no no significant difference in early (OR: 0.39, 95% CI: 0.06 to 2.36, P: 0.30), late (OR: 1.35, 95% CI: 0.78 to 2.33, P: 0.16) or total (OR: 1.20, 95% CI: 0.71 to 2.02, P: 0.17) tunnel or exit-site infections between the 2 groups.

**Conclusion:**

Laparoscopic catheterization and conventional open catheter placement in PD patients have unique advantages, but laparoscopic PD catheterization may be superior to conventional open catheter placement. However, this conclusion needs to be confirmed with further large-sample-size, multi-centre, high-quality RCTs.

## Background

Alternative treatments for end-stage renal disease (ESRD) include kidney transplantation, haemodialysis and peritoneal dialysis (PD). PD has become the preferred alternative treatment for end-stage renal disease because of its low cost, simple technology, strong patient independence, few dietary restrictions, stable haemodynamics and good protection of residual renal function [1]. Although PD is an effective treatment for end-stage renal disease, the success of PD depends on successful PD-catheter. Successful placement of PD catheters can improve the efficiency of PD, reduce complications such as peritonitis and drifting catheters, and prolong the life of patients [2]. Successful placement is also a prerequisite for effective progress in PD. [3] The main operation methods for PD catheterization are traditional operation and laparoscopic operation. The traditional operation for PD catheter insertion involves “blind” insertion of the catheter through a small lower abdominal incision using a malleable catheter guide [4]. This operation has certain blindness, and the operating space is narrow, so it is not easy to accurately locate [5]. Compared with the traditional operation, the laparoscopic operation has the advantages of less trauma and faster recovery after the operation [6], but it requires expensive equipment and specially trained personnel; thus, the procedure is not easily popularized. Several authors [4, 7–10] found a benefit by the addition of laparoscopic guidance, while others [11–14] showed these equivalent in terms of complications and catheter survival.

Because of this argument, a previous meta-analysis compared the complications between laparoscopic placement and conventional insertion of a catheter [15]. The study showed that laparoscopic catheter placement had no superiority to the traditional operation, but only four RCTs were included in the meta-analysis. As several new RCTs have been published recently, an updated meta-analysis is needed to re-evaluate the results.

In this meta-analysis, we systematically reviewed and analysed previous randomized controlled trials to compare the complications from conventional versus laparoscopic catheter placement in PD patients. The results of our study will provide a reference for future methods of PD catheterization.

## Methods

### Search strategy

Three researchers (ZY, ZP and SML) performed a comprehensive literature search, and 8 relevant studies were obtained that conformed to all of the eligible criteria. We searched the electronic databases PubMed, Embase, CNKI, and Cochrane Library for studies published prior to May 6, 2019. The following keywords were used: “Laparoscopic”, “Peritoneal Dialysis”, “Dialysis”, “Conventional”, “Open”, and “Catheter”. Reference lists from the identified studies were included to enrich the analysis.

### Selection criteria

**Fig. 1:**
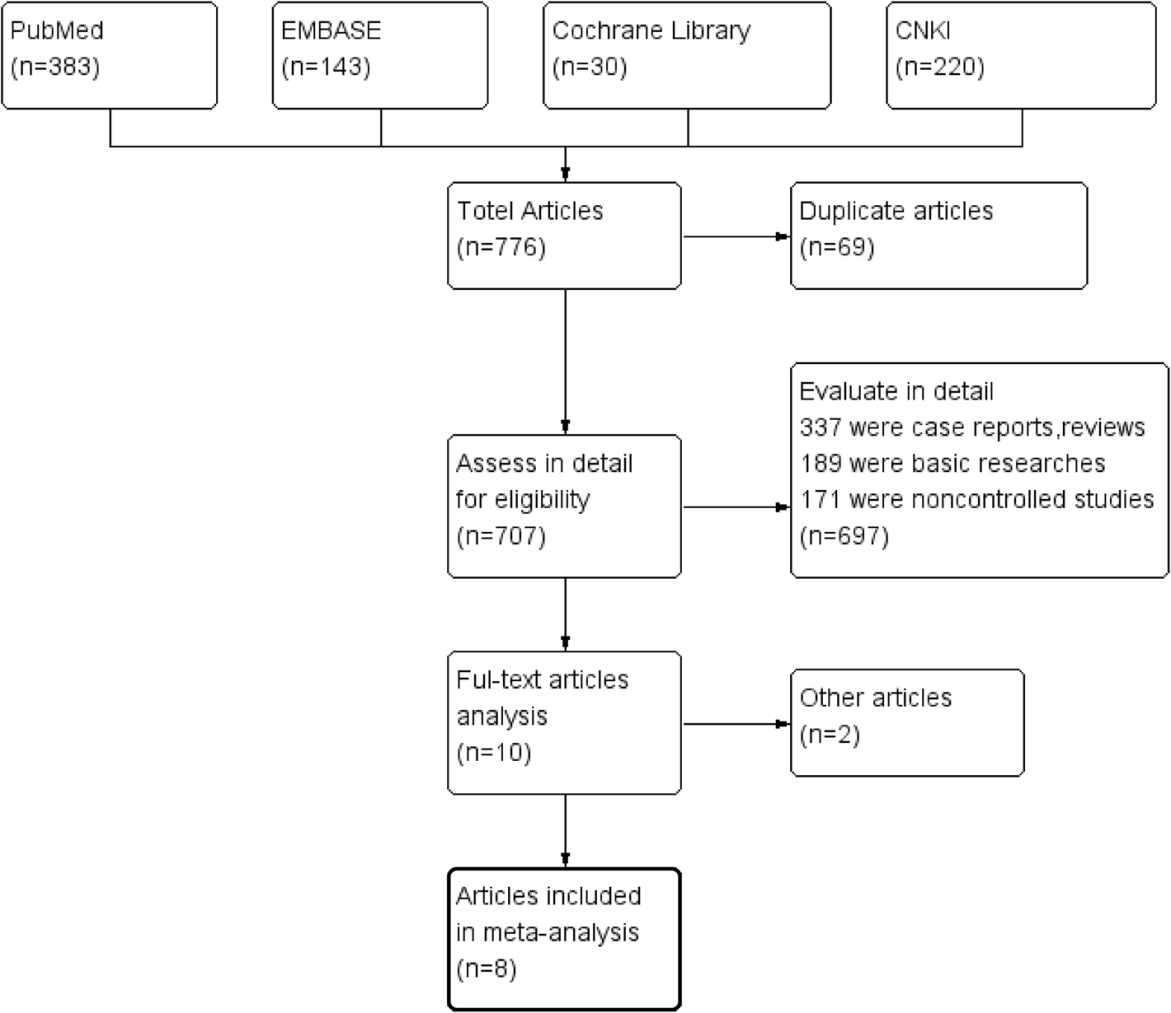
Flow chart of the studies included in the meta-analysis

Three researchers (ZY, ZP and SML) conducted a preliminary review independently to search for randomized controlled trials (RCTs) that met the inclusion criteria. Any discrepancy was resolved by consensus and discussion (Fig. 1). The following criteria were used for inclusion: 1) the study was an RCT; 2) the study compared the outcomes of a laparoscopic PD catheter insertion technique with those of conventional insertion; 3) relative risk (RR) and 95% confidence interval (CI) were calculated; and 4) more than 1 complication was described. The main characteristics of the included studies are listed in Table 1.

**Table 1:** Main characteristics of the included studies

Study	Country	Design	Sample Size (n)		Age (year)		Follow-up (month)		Outcomes
			Laparoscopic	Conventional	Laparoscopic	Conventional	Early	Late	
Gadallah 1999 [16]	USA	RCT	76	72	45.0 ± 1.8	47.2 ± 2.4	0.5	0.5–8	complications
Wright [14] 1999	UK	RCT	24	21	46.4 ± 14.8	49.3 ± 20.2	1.5	1.5–26	complications
Tsimoyiannis 2000 [10]	Greece	RCT	25	25	53.7 ± 12.2	61.4 ± 6.1	36		complications
Jwo 2010 [12]	Taiwan	RCT	37	40	56.6 ± 13.4	54.4 ± 16.5	1	1–8	complications
Laanen 2018 [17]	Netherlands	RCT	46	44	62.6 ± 14.1	64.5 ± 14.1	8		complications
Qiao 2012 [18]	China	RCT	58	58	47.64 ± 13.73		24		complications
Qu 2017 [19]	China	RCT	35	35	39.4 ± 11.3	39.8 ± 11.2	12		complications
Xu 2010 [20]	China	RCT	25	25	53.6 ± 14.6	59.2 ± 16.8	18		complications

### Risk of bias assessment

The quality of all trials was assessed by three authors (ZY, ZP, and SML) independently according to the Cochrane quality criteria (Table 2). Any disagreement was settled by discussion with a fourth author (WB) until a consensus was reached.

**Table 2:** Risk of bias in published randomized control trials

Study	Random sequence generation	Allocation concealment	Blinding of participants and personnel	Blinding of outcome assessment	Incomplete outcome data	Selective reporting	Other bias	Score
Gadallah 1999 [16]	UC	UC	Low risk	Low risk	Low risk (Loss to follow-up: 0)	Low risk	Low risk	5
Wright 1999 [14]	Low risk	Low risk	Low risk	Low risk	Low risk (Loss to follow-up: 0)	Low risk	Low risk	5
Tsimoyiannis 2000 [10]	Low risk	Low risk	Low risk	Low risk	Low risk (Loss to follow-up: 3/50)	Low risk	Low risk	7
Jwo 2010 [12]	UC	UC	Low risk	Low risk	Low risk (Loss to follow-up: 0)	Low risk	Low risk	5
Laanen 2018 [17]	Low risk	Low risk	Low risk	Low risk	Low risk (Loss to follow-up: 5/95)	Low risk	Low risk	7
Qiao 2012 [18]	UC	UC	Low risk	Low risk	Low risk (Loss to follow-up: 6/116)	Low risk	Low risk	5
Qu 2017 [19]	UC	UC	Low risk	Low risk	Low risk (Loss to follow-up: 0)	Low risk	Low risk	5
Xu 2010 [20]	UC	UC	Low risk	Low risk	Low risk (Loss to follow-up: 0)	Low risk	Low risk	5

*UC* unclear

### Statistical analysis

Revman 5.3 software was used to perform the statistical analyses. The odds ratio (OR) with its 95% confidence interval (CI) was used for dichotomous data. If there was no significant heterogeneity, a weighted fixed-effect model was used. Otherwise, a random-effects model was used [21]. Heterogeneity was analysed statistically by the I^2^ and Chi^2^ statistics. The critical value for homogeneity was a *P* value less than 0.05. A sensitivity analysis was conducted by omitting each study in turn to evaluate the quality and consistency of the results.

Heterogeneity was determined as follows: an Ι^2^ statistic of 0 to 25% was considered low heterogeneity; 25 to 50% was medium heterogeneity; 50 to 75% was high heterogeneity; and 75 to 100% was considered powerful heterogeneity. The *P* value was determined using the χ2 test; it was considered statistically significant when *P* < 0.05 [22].

## Results

### Study selection

We identified a total of 776 articles in the initial retrieval. In this study, 69 duplicate articles were discarded after carefully reviewing the titles and abstracts. When evaluated in detail, 697 articles were excluded because 189 were basic research studies, 171 were non-controlled studies, and 337 were case reports or reviews. The remaining 10 articles were reviewed for a more detailed assessment. An additional 2 articles were excluded due to a lack of available data. Finally, 8 studies with 646 participants fulfilled the inclusion criteria for this meta-analysis**.** The main characteristics of the included RCTs (country, sample size, design, patient age, intervention and follow-up) are described in Table 1. The retrieval strategy is described in the flow diagram (Fig. 1).

### Sensitivity analysis and publication bias

No significant heterogeneity was found in the results. Sensitivity analysis was performed to evaluate the stability of our results. The results showed that no individual studies significantly affected the heterogeneity.

### Catheter-related complications

#### Migration

**Fig. 2:**
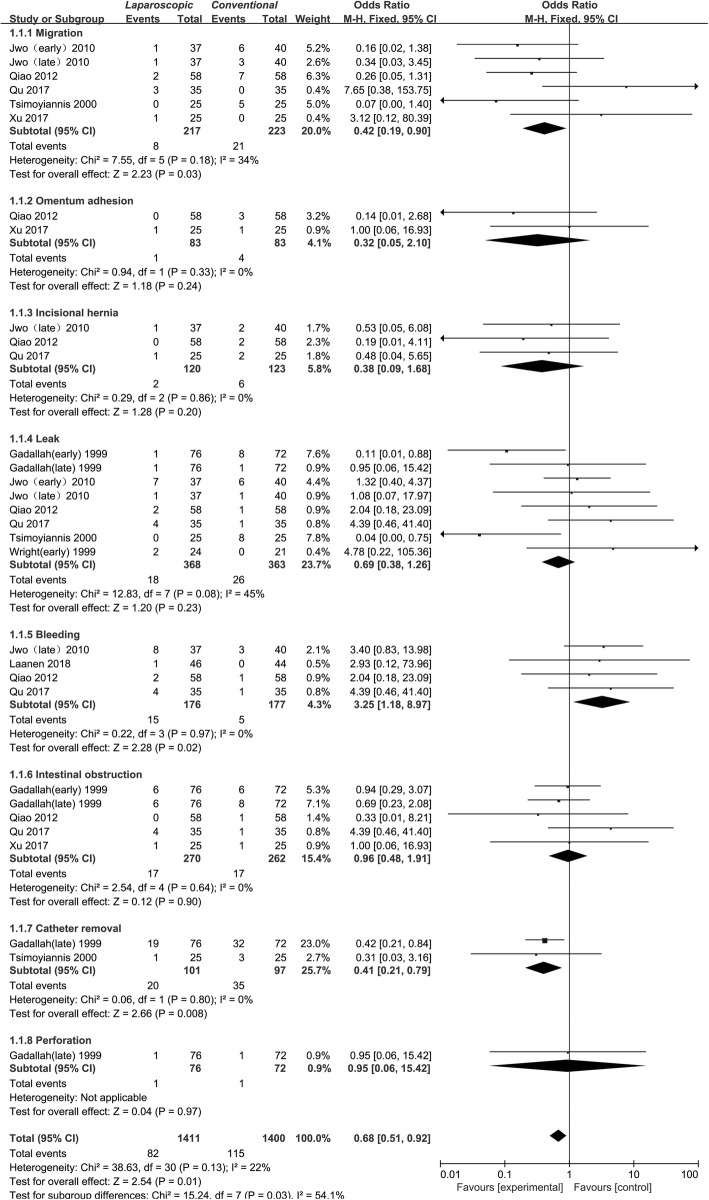
Forest plot of catheter-related complications in observational studies. The follow-up times of “early” and “late” are defined in Table 2

Five studies [10, 12, 18–20] assessed catheter migration in a total of 440per patient-years, with 217 assigned to laparoscopic groups and 223 assigned to conventional open groups. Because there was no significant heterogeneity, the fixed-effects model was used (I^2^ = 34%). The statistical analysis showed a lower incidence of catheter migration in the laparoscopic group than in the conventional open group (OR: 0.42, 95% CI: 0.19 to 0.90, P: 0.03), as presented in Table 3 and Fig. 2.

**Table 3:** The results of catheter-related complications in observational studies

Infections		Results				Heterogeneity		
Parameter	N/Pt-yr	Effects Model	Pooled Estimate	95% CI	*P*-value	Chi^2^	I^2^	P-value
Migration [10, 12, 18–20]	5/440	Fixed	0.42	0.19 to 0.90	0.03	7.55	34%	0.18
Omentum adhesion [18, 20]	2/166	Fixed	0.32	0.05 to 2.10	0.24	0.94	0%	0.33
Hernia [12, 18, 19]	3/243	Fixed	0.38	0.09 to 1.68	0.20	0.29	0%	0.20
Leakage [10, 12, 14, 16, 18, 19]	8/731	Fixed	0.69	0.38 to 1.26	0.23	12.83	45%	0.08
Bleeding [12, 17–19]	4/263	Fixed	3.88	1.28 to 11.77	0.02	0.08	0%	0.96
Intestinal obstruction [16, 18–20]	5/532	Fixed	0.96	0.48 to 1.91	0.90	2.54	0%	0.64
Catheter removal [10, 16]	2/198	Fixed	0.41	0.21 to 0.79	0.008	0.06	0%	0.80
Perforation [16]	1/148	Fixed	0.95	0.06 to 15.42	0.97	Not applicable		

N/Pt-yr: No. of studies/Patient-years

#### Omentum adhesion

Only 2 studies [18, 20] reported an incidence of omentum adhesion involving 166 per patient-years, with 83 assigned to laparoscopic groups and 83 assigned toconventional open groups. Because there was no significant heterogeneity, the fixed-effects model was used (I^2^ = 0%). The statistical analysis showed no significant difference between the 2 groups (OR: 0.32, 95% CI: 0.05 to 2.10, P: 0.24), as presented in Table 3 and Fig. 2.

#### Hernia

Three studies [12, 18, 19] assessed the incidence of incisional hernias in a total of 243 per patient-years, with 120 assigned to laparoscopic groups and 123 to conventional open groups. Because there was no significant heterogeneity, the fixed-effects model was used (I^2^ = 0%). The statistical analysis showed no significant difference between the 2 groups (OR: 0.38, 95% CI: 0.09 to 1.68, P: 0.20), as presented in Table 3 and Fig. 2.

#### Leakage

The incidence of leakage was reported in 6 studies [10, 12, 14, 16, 18, 19] involving 731 per patient-years, with 368 assigned to laparoscopic groups and 363 assigned to conventional open groups. Because there was no significant heterogeneity, the fixed-effects model was used (I^2^ = 45%). The statistical analysis showed no significant difference between the 2 groups (P: 0.23, OR: 0.69, 95% CI: 0.38 to 1.26), as presented in Table 3 and Fig. 2.

#### Bleeding

The incidence of bleeding was reported in 4 studies [12, 17–19] involving 353 per patient-years, with 176 assigned to laparoscopic groups and 177 assigned to conventional open groups. Because there was no significant heterogeneity, the fixed-effects model was used (I^2^ = 0%). Compared with patients in the conventional open groups, patients in the laparoscopic groups showed a statistically significant increase in the incidence of bleeding ((OR: 3.25, 95% CI: 1.18 to 8.97, P: 0.02), as assigned in Table 3 and Fig. 2.

#### Intestinal obstruction

Four studies [16, 18–20] assessed the incidence of intestinal obstruction in a total of 532 per patient-years, with 270 assigned to laparoscopic groups and 262 assigned to conventional open groups. Because there was no significant heterogeneity, the fixed-effects model was used (I^2^ = 0%). The statistical analysis showed no significant difference between the 2 groups (OR: 0.96, 95% CI: 0.48 to 1.91, P: 0.90), as presented in Table 3 and Fig. 2.

#### Catheter removal

Only 2 studies [10, 16] reported the incidence of catheter removal in a total of 198 per patient-years, with 101 assigned to laparoscopic groups and 97 assigned to conventional open groups. Because there was no significant heterogeneity, the fixed-effects model was used (I^2^ = 0%). The statistical analysis showed a lower incidence of catheter removal in the laparoscopic group than in the conventional open group (OR: 0.41, 95% CI: 0.21 to 0.79, P: 0.008), as presented in Table 3 and Fig. 2.

#### Perforation

Only 1 study [16] reported the incidence of perforation, and it involved 148 per patient-years, with 76 assigned to laparoscopic groups and 72 assigned to conventional open groups. Because there was only 1 study describing perforation, heterogeneity analysis was not applicable. The statistical analysis showed no significant difference between the 2 groups (OR: 0.95, 95% CI: 0.06 to 15.42, P: 0.97), as presented in Table 3 and Fig. 2.

### Infections

Infections were divided into “early” and “late”. The definitions of “early” and “late” are shown in Table 1. Early infections are usually related to catheter placement; late infections are usually related to multiple factors other than the surgical procedure [12]. A measure of early and late infections was reported in 3 [12, 14, 16] of the 8 trials.

#### Peritonitis

**Fig. 3:**
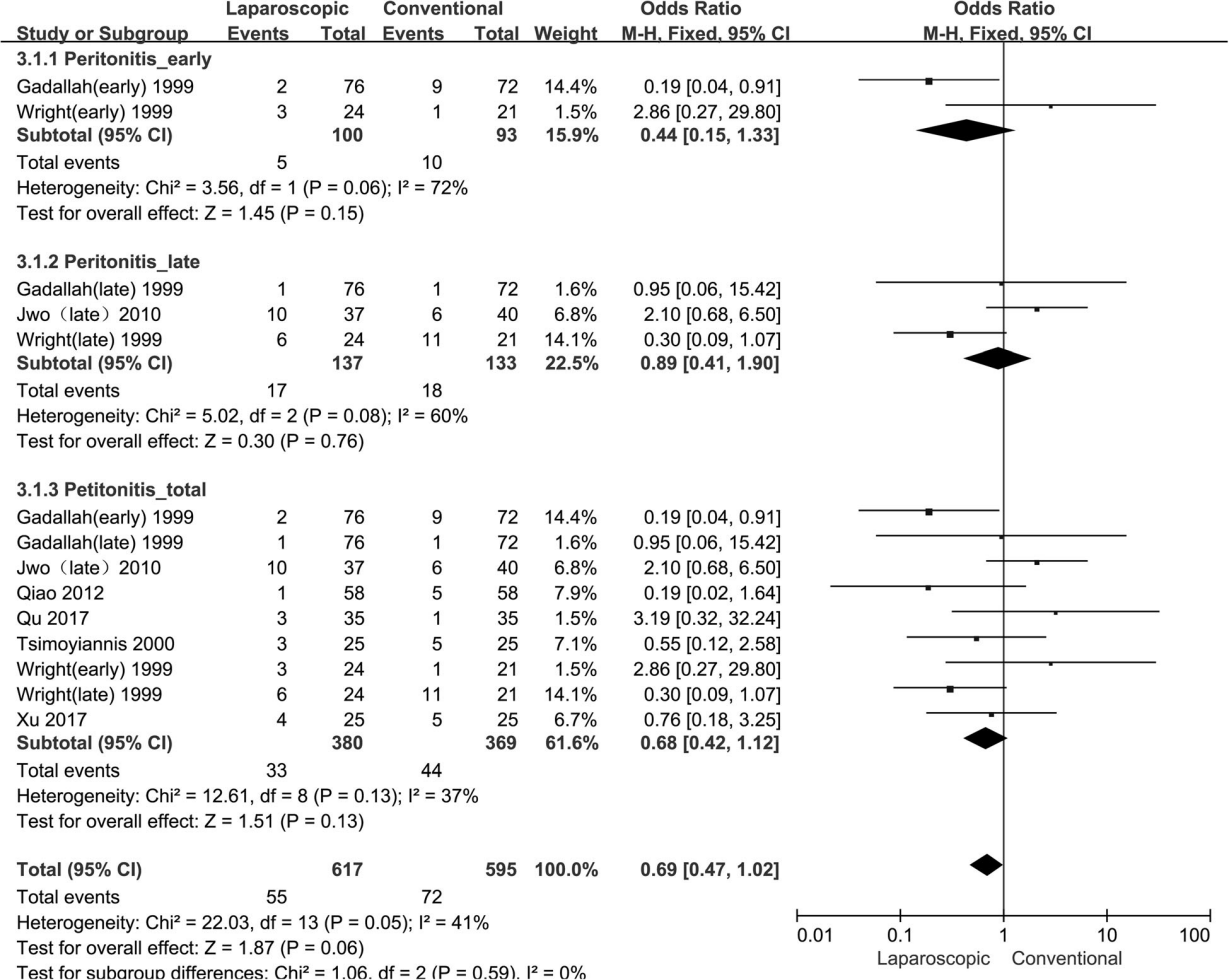
Forest plot of peritonitis in observational studies. The follow-up times of “early” and “late” are defined in Table 2

Seven studies [10, 12, 14, 16, 18–20] assessed the incidence of peritonitis in a total of 749 per patient-years. Because there was no significant heterogeneity, the fixed-effects model was used (I^2^ = 41%). The statistical analysis showed no significant difference in early (OR: 0.44, 95% CI: 0.15 to 1.33, P: 0.15), late (OR: 0.89, 95% CI: 0.41 to 1.90, P: 0.76) or total (OR: 0.68, 95% CI: 0.42 to 1.12, P: 0.13) peritonitis infections between the 2 groups, as presented in Table 4 and Fig. 3.

**Table 4:** The results of infections in observational studies

Infections		Results				Heterogeneity		
Parameter	N/Pt-yr	Effects Model	Pooled Estimate	95% CI	P-value	Chi^2^	I^2^	P-value
Peritonitis								
Early [14, 16]	2/193	fixed	0.44	0.15 to 1.33	0.15	3.56	72%	0.15
Late [12, 14, 16]	3/270	fixed	0.89	0.41 to 1.90	0.76	5.02	60%	0.76
Total [10, 12, 14, 16, 18–20]	9/749	fixed	0.68	0.42 to 1.12	0.13	12.61	37%	0.13
Tunnel /exit-site infection								
Early [14]	1/45	fixed	0.39	0.06 to 2.36	0.30	Not applicable		
Late [12, 14, 16]	3/270	fixed	1.35	0.78 to 2.33	0.16	3.68	46%	0.29
Total [12, 14, 16]	4/315	fixed	1.20	0.71 to 2.02	0.49	5.00	40%	0.17

N/Pt-yr: No. of studies / Patient-years

**Fig. 4:**
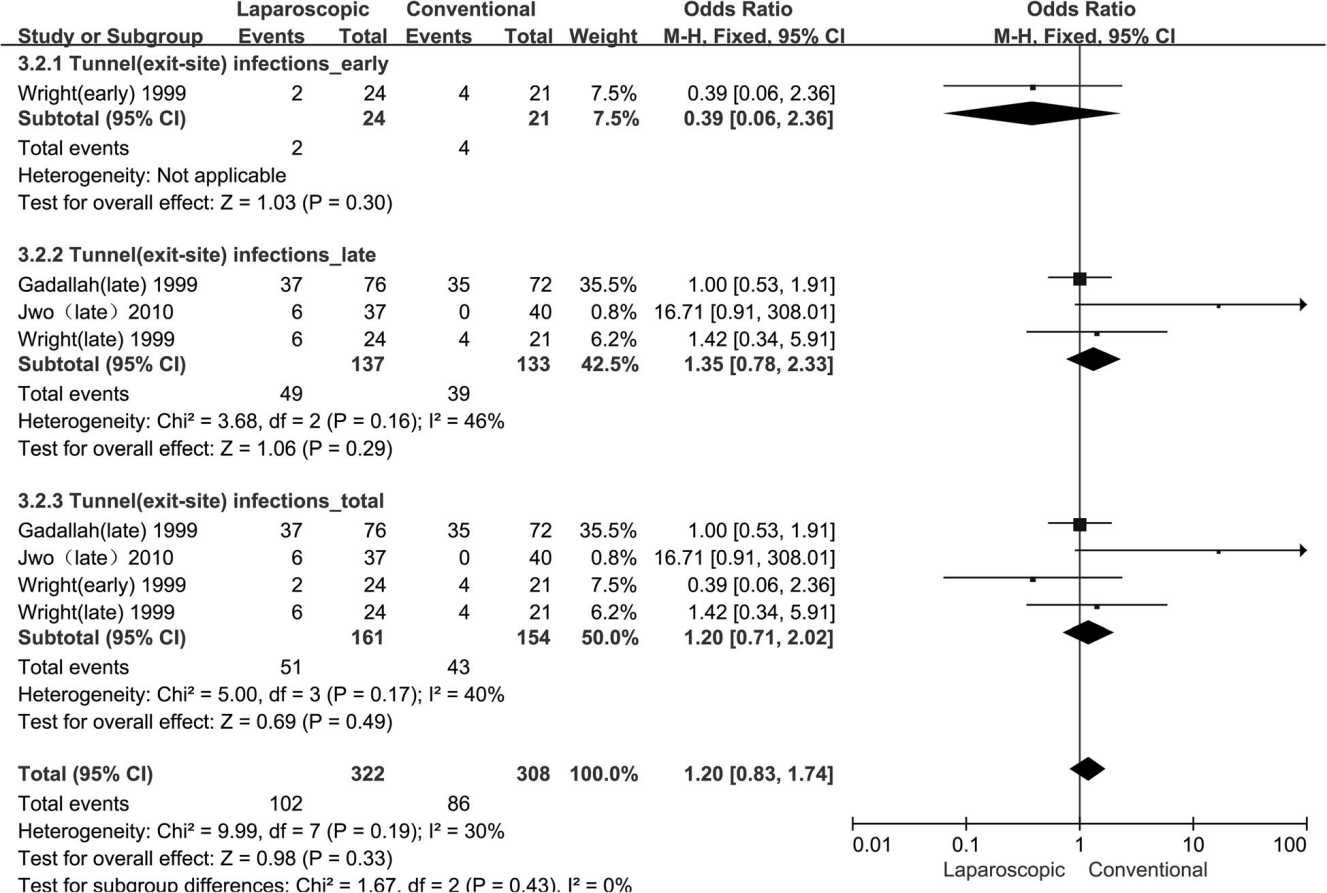
Forest plot of tunnel (exit-site) infections in observational studies. The follow-up times of “early” and “late” are defined in Table 2

Tunnel or exit-site infections: Three studies [12, 14, 16] assessed the incidence of tunnel or exit-site infections in a total of 315 per patient-years. Because there was no significant heterogeneity, the fixed-effects model was used (I^2^ = 30%). The statistical analysis showed no significant difference in early (OR: 0.39, 95% CI: 0.06 to 2.36, P: 0.30), late (OR: 1.35, 95% CI: 0.78 to 2.33, P: 0.16) or total (OR: 1.20, 95% CI: 0.71 to 2.02, P: 0.17) tunnel or exit-site infections between the 2 groups, as presented in Table 4 and Fig. 4.

## Discussion

In 1959, Richard Ruben [23] successfully used peritoneal dialysis (PD) for the first time. Popovich and Moncrief developed continuous ambulatory PD, which promoted the use of PD. [24] Subsequently, the method of introducing catheters into the abdominal cavity was modified, and then an open operation, percutaneous puncture, peritoneoscopy and laparoscopic techniques were introduced [14, 25, 26]. Several authors favour laparoscopic catheter placement over conventional surgery and demonstrate the obvious advantages of laparoscopic PD-catheter in non-randomized trials [4, 9, 27]. However, due to the lack of RCTs with high quality and large sample sizes, this conclusion is still controversial.

Recently, a few RCTs examining the two techniques have been published. Gadallah et al. conducted an RCT with 148 patients addressing the use of the laparoscope for dialysis catheter implantation and provided us with some suggestions for catheter placement [16]. Later, Jwo et al. conducted an RCT with 77 patients for comparison of conventional placement with laparoscopic-assisted placement of a Tenckhoff PD catheter; they wrote a report and concluded that laparoscopic-assisted catheter placement exhibited no superiority to the conventional operation technique [12]. A previous meta-analysis compared the two surgical methods. They also found that laparoscopic catheter placement had no superiority to the traditional operation, but only four RCTs were included in the meta-analysis. As several new RCTs have been published recently, a number of different views have emerged. Therefore, we performed a meta-analysis to make it convenient for clinicians to select the appropriate surgical approach.

In this study, we conducted a meta-analysis to compare the complications of laparoscopic versus conventional catheter placement in PD patients. Our results showed that laparoscopic insertion could significantly decrease the probability of migration and catheter removal. However, laparoscopic PD-catheter has a higher risk of bleeding than conventional open catheterization. Jwo et al. reported that the higher incidence of bleeding in the laparoscopic group may be due to the fact that the puncture procedure lacks sufficient monitoring of bleeding [12]. No significant difference was found in other complications, such as omentum adhesion, hernia, leakage, intestinal obstruction and perforation. No statistically significant difference was found in the incidence of peritonitis or exit-site/tunnel infection.

Compared with laparoscopic minimally invasive PD catheterization, conventional PD catheterization has the following disadvantages: 1) a long operation time, strong pain (laparoscopy PD catheterization is a general anesthesia operation, while conventional PD catheterization is under local or epidural anesthesia. Therefore, patients often have “strong pain” during the conventional PD catheterization), a long incision length and a slow recovery [28]; 2) a limited field of vision, as it is not as open as the laparoscopic minimally invasive operation, and blindcatheter placement by hand leads to inaccurate catheter placement, easy catheter movement or catheter obstruction by the greater omentum [3]; 3) the incision infection rate is high, with ESRD often associated with a variety of diseases, poor resistance, and traditional PD catheterization associated with a longer incision length and a high infection rate that often lead to surgical failure [3]; and 4) the operation is more difficult for obese patients [3]. Zhang et al. reported that the failure rate of conventional open PD catheter placement could reach 10.0 to 22.0%. Therefore, accurate intraoperative positioning and fixation and prevention of postoperative infection are important for successful PD treatment.

Lee et al. reported [29] that 102 patients who received PD were divided into two groups, which received either laparoscopic or conventional catheter placement, and were followed up for 6 months after the operation. The results showed that the probability of transabdominal tube displacement and blockage in patients who received traditional laparotomy was 12%. However, no drift or blockage of the peritoneal tube occurred in patients undergoing laparoscopic peritoneal catheterization. There are other reports of laparoscopic PD catheter placement describing excellent results. Ko J et al. reported [30] that the success rate of laparoscopic PD-catheter was 100%. Other researchers have used stitches to fix the catheter in place during laparoscopy, with reported success rates of 94 to 100% [27, 31]. A study by Ko et al. also showed a favourable outcome when fixing the catheter to the lower abdominal wall. In their report, only 1 late migration (2.6%) of the catheter occurred. Regretfully, patient details were not provided in the study [30].

There are several limitations of our meta-analysis that should be taken into account. First, the information in several studies was incomplete because of the lack of sufficient data, and subgroup analysis based on study type or study region was not conducted. Second, the follow-up times in some studies were different and could have affected our conclusions. Third, as mentioned in the individual studies, the conditions and techniques investigated in the studies varied widely. The RCTs in this meta-analysis had key methodological limitations, particularly due to participant attrition and unclear blinding methods, which reduced our confidence in the conclusions drawn from the contributing data. Finally, the inevitable result of these practice trials was that there were so many laparoscopic techniques used by surgeons for catheter placement, and these different PD-catheter techniques may have affected the final results. Despite these limitations, our results are very meaningful for understanding the differences in outcomes between laparoscopic catheter placement and conventional open operation catheter placement. These limitations also encourage researchers to design stricter RCTs in the future.

## Conclusions

Qie et al. reported [1] that PD-catheter related complications such as catheter migration and catheter removal are the common causes of technical failure of peritoneal dialysis, our meta-analysis demonstrates that compared with conventional open PD-catheter placement, laparoscopic catheterization can reduce the occurrence of catheter migration and catheter removal. Therefore, we believe that the laproscopic PD catheter may be superior to conventional open catheter placement, especially for those patients with abdominal surgery history who are not suitable for open surgery [18]. However, laparoscopic PD-catheter has a higher risk of bleeding than conventional open PD-catheter. Our results will provide a reference for choosing a PD catheter placement technique.

## Data Availability

All data generated or analysed during this study are included in this published article.

## Abbreviations

CI: Confidence interval
CNKI: China National Knowledge Infrastructure database
ESRD: End-stage renal disease
OR: Odds ratio
PD: Peritoneal dialysis
RCT: Randomized controlled trial
